# Effects of nurses-led multidisciplinary-based psychological management in spinal surgery: a retrospective, propensity-score-matching comparative study

**DOI:** 10.1186/s12912-024-01842-y

**Published:** 2024-03-29

**Authors:** Ying Liu, Jiali Chen, Tingkui Wu, Junbo He, Beiyu Wang, Peifang Li, Ning Ning, Hong Chen

**Affiliations:** 1 Department of Orthopedic Surgery, West China Hospital, Sichuan University/School of Nursing, Sichuan University, No. 37 Guoxue Xiang, Chengdu, Sichuan 610041 P.R. China; 2 Faculty of Nursing, Midwifery and Palliative Care, King’s College London, London, UK; 3 Department of Orthopedic Surgery, West China Hospital, Sichuan University, No. 37 Guoxue Xiang, Chengdu, Sichuan 610041 P.R. China; 4 West China School of Nursing, West China Hospital, Sichuan University, No. 37 Guoxue Xiang, Chengdu, Sichuan 610041 P.R. China

**Keywords:** Emotional disorders, Nurses-led management, Perioperative Period, Propensity score matching, Psychosocial intervention, Retrospective studies, Spine surgery

## Abstract

**Background:**

Patients in spine surgery often have emotional disorders which is caused by multi-factors. Therefore, a multidisciplinary and multimodal intervention program is required to improve emotional disorders during the perioperative period. However, related studies were rare. This study aimed to confirm that the multidisciplinary-based psychological management leading by nurses was effective in treating emotional disorders and show the assignments of the members of the multidisciplinary team with the orientations of nurses.

**Design:**

A retrospective, comparative study.

**Method:**

This study was a retrospective cohort research and compared the results between the intervention group and control group using the Huaxi Emotional Distress Index (HEI) which was used to evaluate emotional disorders. The intervention group consisted of patients who underwent surgery between January 2018 and December 2020 after psychological management was implemented. The control group consisted of patients with regular care who underwent surgery between January 2015 and December 2017. To improve comparability between the two groups, baseline data from the recruited patients were analyzed using propensity-score-matching (PSM) based on age, sex, marital status, education, and disease region.

**Results:**

A total of 539 (11.5%) people developed emotional disorders, of which 319 (6.8%), 151 (3.2%) and 69 (1.5%) had mild, moderate mood and severe emotional disorders, respectively. 2107 pairs of patients were matched after PSM. Scores of HEI in the intervention group were heightened compared with those in the control group (*P*<0.001) after matching. Moreover, the incidence of emotional disorders in patients decreased after implementing psychological management (*P* = 0.001). The severity of emotional disorders was alleviated with statistical significance as well (*P* = 0.010).

**Conclusions:**

Nurses-led Multidisciplinary-Based psychological management was able to reduce the incidence of emotional disorders and improve the severity of these in spine surgery patients.

**Supplementary Information:**

The online version contains supplementary material available at 10.1186/s12912-024-01842-y.

## Introduction

Spine degenerative diseases are highly prevalent worldwide and bring about a huge economic burden [1]. It estimated that direct costs were more than $80 billion for treatment of them [2, 3]. Spine surgery reduces patients’ suffering in degenerative spine conditions such as degenerative lumbar stenosis, disc herniation, and degenerative scoliosis [4–6]. However, surgery is a stressful experience for most people. A great deal of patients experienced emotional disorders, with 1 out of 3 experiencing depression or anxiety [7]. According to a cross-sectional study of 512 patients undergoing spine surgery, 55% had preoperative anxiety, and 54% had preoperative depression [8]. Emotional disorders such as anxiety might lead to exacerbated functional outcomes and clinical prognoses [9, 10]. Therefore, effective psychological management is beneficial to the prognosis of patients with spinal surgery.

Perioperative psychological management for spine surgery, such as pain management and timely psychological or information support, has been reported [7, 11]. However, these management schemes are unilateral, and emotional disorders are factorial [12]. It is well documented that patients with pain and multimorbidity are more prone to depression than the general population [13, 14]. Other studies have also found that the level of disability and knowledge of disease information affected the level of emotional disorders in patients with spine surgery [12]. Therefore, perioperative psychological management requires the cooperation of a multidisciplinary team. In 1997, Henrik Kehlet proposed a multimodal approach to perioperative management known as “Fast-Track Surgery” [15]. This approach was developed into the Enhanced Postoperative Recovery (ERAS) program aimed at improving recovery, which is an evidence-based approach to perioperative care and related to anesthesia, nutrition, sleep and other aspects [16]. Improving surgical outcomes, reducing complications, improving patient experience, and reducing hospital stays are the main goals of ERAS [17, 18]. Under the concept of ERAS, a multidisciplinary team is needed to boost a certain clinical outcome during the perioperative period [19]. Previously, numerous studies have confirmed the ERAS protocols in perioperative management effectively [20]. Nevertheless, multidisciplinary-based psychological management in spine surgery patients is still controversial.

Hence, this study sought to investigate the prevalence of emotional disorders and evaluate whether multidisciplinary-based psychological management was effective in improving emotional disorders in patients with spine surgery. This study discussed how multidisciplinary teams collaborate and the way nurses leading to.

## Article types

Original research.

## Materials and methods

### Study design

The study was designed as a nonrandomized controlled clinical trial. With the intent of comparing the incidence of emotional disorders and the differences in the composition of grades in emotional disorders of patients before and after the implementation of the management, we conducted a retrospective evaluation between January 2015 and December 2020 in a single-center orthopedic spine department. Baseline data of the enrolled patients were analyzed using propensity-score-matching (PSM) to enhance comparability between the two groups. Our study was conducted in accordance to the relevant guidelines and regulations or in accordance to the Declaration of Helsinki. The study protocol was reviewed and approved by the Research Ethics Committee of the Institutional Review Board (2017 (Review) No. 128). This study was conducted in accordance to the relevant guidelines and regulations.

### Patient sample

The inclusion criteria for patients were as follows: (1) age greater than 16 years old, (2) surgery scheduled for the first time, and (3) had spine surgery. Exclusion criteria included trauma, infection, and cancer as indications for surgeries. Patients who were diagnosed with sleep disturbances were not considered. The intervention group consisted of patients who underwent surgery between January 2018 and December 2020 after psychological management was implemented. The controls were matched with those who underwent surgery prior to initiation of psychological management based on age, sex, marital status, education, and disease region in the same medical unit. Therefore, the control consisted of patients with regular care who underwent surgery between January 2015 and December 2017.

### Outcome measures

Anxiety, depression, suicidal ideation, and other psychological problems, known as emotional disorders, were measured by the Huaxi Emotional-distress Index (HEI) [21]. The scale has a sensitivity of 88% and a specificity of 76.58%. The internal consistency was 0.898. The HEI self-evaluation scale was developed by West China Hospital of Sichuan University for the rapid screening of patients’ psychological status in nonpsychiatric clinical settings. The main scale consists of nine items that can be finished in less than 5 min. Using the 5-point Likert-scale scoring method, the total score is 36. Scores ranging from 0 to 8 indicate normal or no emotional disorder. Scores of 9 to 12 represent mild emotional disorder. Ranging from 13 to 16 hints at moderate emotional disorder, and over 17 represents severe emotional disorder. Items 10 and 11 were used as references and only appeared when items 1–9 scored more than 9 points and were not included in the final score. Patients who received a HEI score greater than 8 points were perceived as having emotional disorders. In this study, the postoperatively measured HEI score was used to represent the level of perioperative emotional disorders of patients.

### Psychological management program

Under the guidance of a multidisciplinary team of experts, management based on ERAS was established in the spine surgery ward in 2017. This model focused on the improvement of patients’ psychology, sleep, rehabilitation and other aspects. A psychological management program for patients has been implemented since January 2018. During the implementation process, the content of the program was adjusted in time. Regular and complete psychological management was carried out finally.

**Establishment of a multidisciplinary team (MDT)** According to Best Evidence Summary, the core members’ departments were confirmed, including orthopedics, mental health centers, anesthesiology and pharmacy [22]. The medical team that closely cooperated with the orthopedic department was selected as a member of the cooperative management group from the included departments. Sunshine Angels, orthopedic nurses who have obtained the qualifications of psychological consultants and get the training from the Mental Health Center, took responsibility for psychological assessment and basic supports for patients directly. Additionally, she would feed back the general emotional situation of patients, interventions, effects of interventions and other information to all levels of management, who decided on further intervention programs. Under the leadership of the multidisciplinary experts from management group, the implementation of specific measures was progressive from the decision-making layer (medical team leader, head nurse) to the implementation layer (resident surgeons, advanced doctors, nursing team leaders, charge nurses) and then to the security layer (mental health experts, anesthesiology experts, pharmacy experts). Table 1 revealed the members’ tasks in detail.

**Table 1:** Responsibilities of the multidisciplinary psychological management group

Member	Responsibilities
Orthopedic surgeon	① Fully responsible for the safety management of patients during the perioperative period ② Minimally invasive surgery, shorten the operation time and reduce the stress of surgery ③ Carry out intervention treatment according to the dynamic condition of the patient
“Sunshine angel” (nurse)	①Use HEI to evaluate the psychological of patients ②Implement bedside intervention (coaching, communication, and relaxation training) for patients with mild to moderate emotional disorders ③ Communicate and supervise the implementation of work among members of the group, and regularly feedback the overall work progress
Mental physician	①Lead the development of psychological counseling programs ②Implement specialized cognitive-behavioral interventions
Anesthesiologist	①Standard anesthesia; ②Active analgesia ③Individualized analgesia
Pharmacist	① Review the rationality of drug use ② Regularly carry out standardized drug use training and update the pharmacological knowledge reserve of core members of the team

**Fig. 1:**
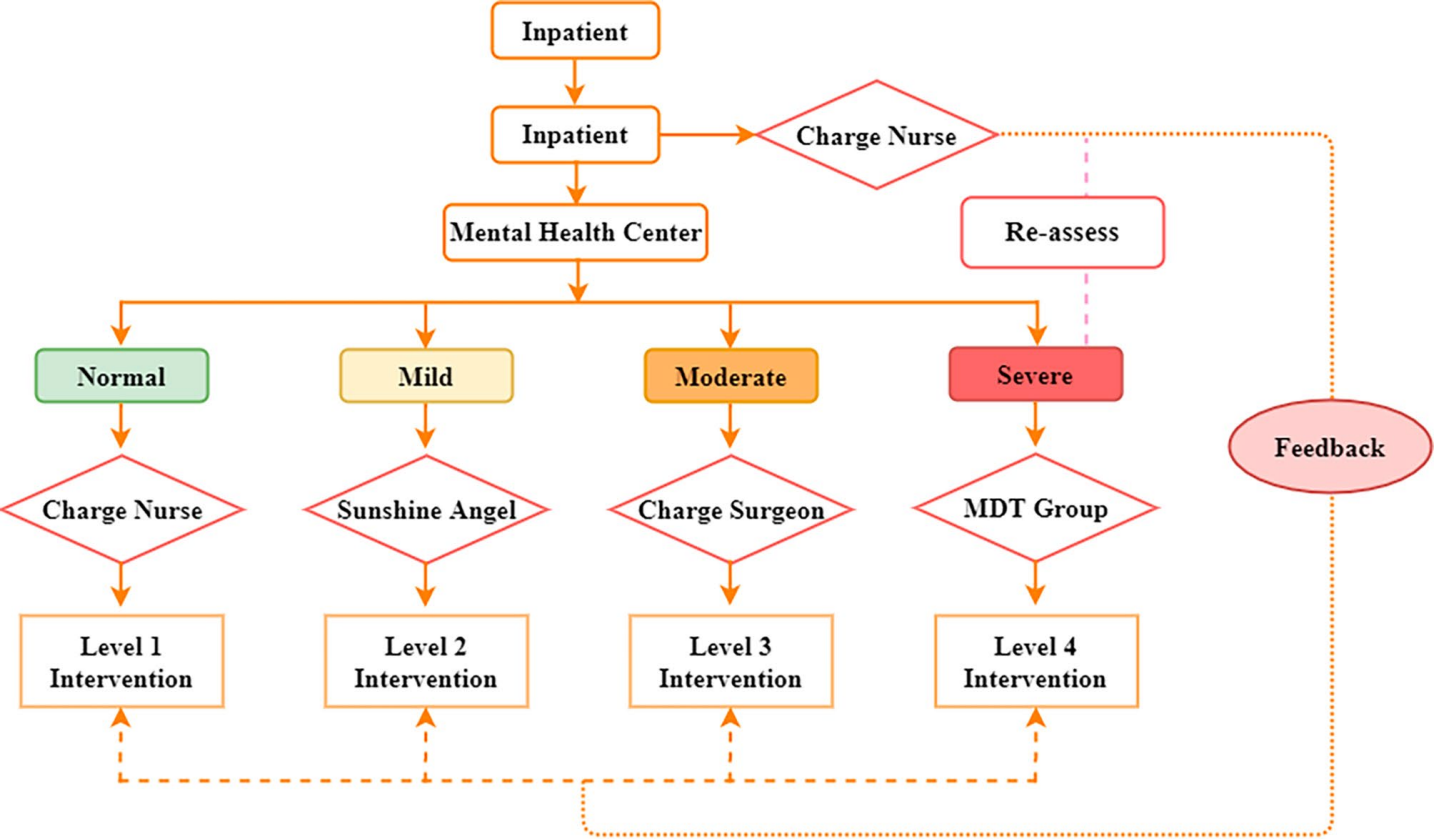
Pathway of multidisciplinary-based psychological management

**Regular pathway of psychological management** Fig. 1 illustrated the flow diagram of the management pathway. All patients were evaluated for HEI within 2 h of admission by the nurses in charge. In the case of a patient with a severe emotional disorder, an additional assessment within 24 h was needed. Results of HEI were registered and fed back by the mental health center who reported patients with emotional disorders to the ward. The surgeon in charge would receive the results of the psychological assessment from the patient’s nurse in charge as soon as possible. Then, the orthopedic ward formulated corresponding psychological management according to the level of emotional disorders. Multidisciplinary-Based Psychological Management adopted a 4-level stepwise intervention for patients.

- Level 1 (charge nurses): The main task is to provide simple psychological support to the patient. The charge nurses used their interpersonal skills to ask patients whether they needed assistance. They tended to encourage patients who have not yet manifested emotional illnesses by listening to them and showing empathy.
- Level 2 (sunshine-angel-led physical prevention): The Sunshine Angel had to step in again when patients developed emotional disorders. The first time the reasons of the patient’s emotional disorders were recognized was through a professional and quick psychological consultation. Sunshine Angels only offered psychological supports through physical means due to they are not permitted to prescribe in China. Music therapy, which may help patients learn to relax and treat emotional issues, was the physical technique employed in this study (Solanki et al., 2013).
- Level 3 (Surgeon-led medication prevention): Physical therapies were ineffective when patients had mild emotional difficulties. In order to treat emotional problems, combination medications were therefore required. A mixture of analgesics (nonsteroidal anti-inflammatory medicines), anti-anxiety medications (paroxetine hydrochloride), and sleep aids (zolpidem tartrate, estazolam) was utilized as the foundation for the Level 2 intervention. The medication must be taken in accordance with a doctor’s prescription, and nurses were present to ensure that the patients took their medications on schedule.
- Level 4 (MDT-led integrated intervention): MDT-led integrated therapies were necessary when patients had serious emotional problems. Early intervention by medical professionals from the mental health facility included professional behavior and cognitive therapy paired with medication, as well as extensive cognitive behavioral therapy and relaxation treatment. Patients were expected to take their medications as directed by their doctors, supervised by charge nurses. If required, the patient was sent to mental care after their specialized condition was stabilized.

### Statistical analysis

Statistical description was performed using Python 3.9.5 software, and statistical analyses were performed using SPSS 22.0 software((SPSS, Chicago, IL, USA)with the PSM plug-in. The characteristics of patients and the incidence of emotional disorders were displayed using a heatmap. To control for bias, we used PSM by the Nearest Neighbor Matching method with a ratio of 1:1. Age, sex, marital status, education level, and disease region were conducted as covariance of matching by 3-step procedure: development of propensity score, matching, and assessment imbalance. Logistic regression was used to calculate propensity scores, that is, to predict the likelihood of entry into the group implementing psychological management based on ERAS. Matching was performed by a greedy algorithm, and the caliper distance was 0.02 with exact matching and fuzzy matching. For continuous variables, the standard mean deviation (SMD) was used to assess the balance after matching. SMD of the categorical variable was transformed by the formula and used for the balance test as well. SMD < 0.02 was considered a small effect size. The McNemar test was used for matched binary variables, and the chi-square test was used for unordered multi-categorical variables for analysis. Rank tests were used for ordinal multi-categorical variables and nonnormal continuous variables. The paired *t* test was used for normal continuous variables after PSM. The test level was *P* < 0.05.

## Results

### General information of patients

**Fig. 2:**
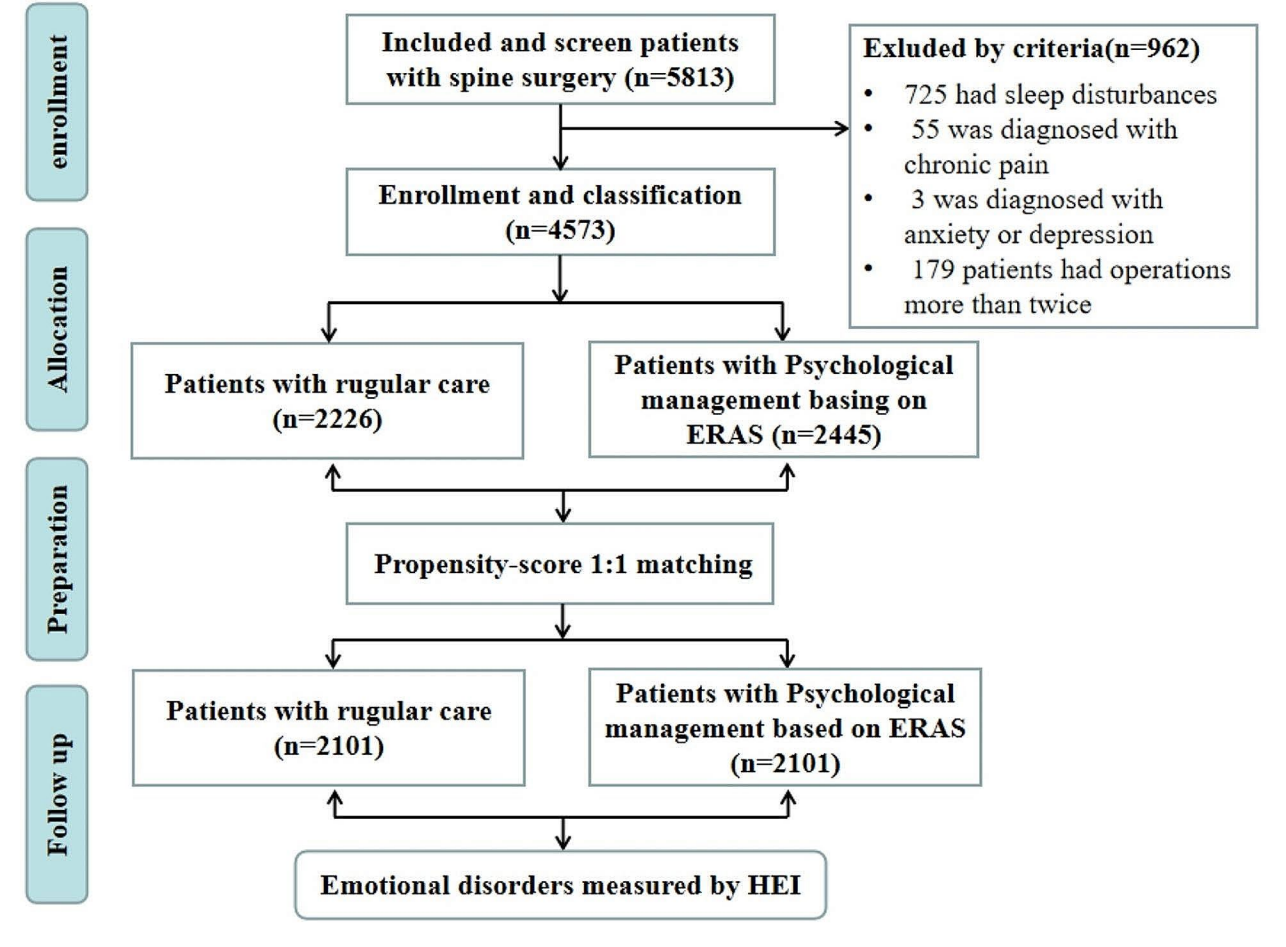
Flow diagram of the study

**Fig. 3:**
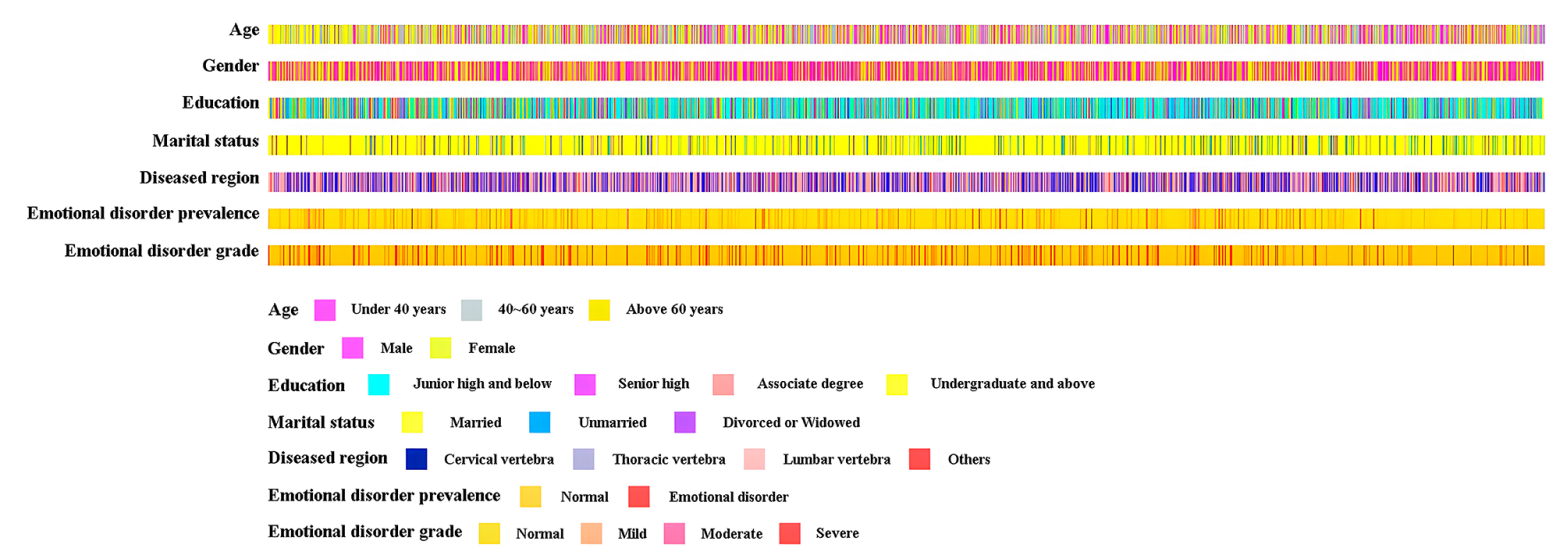
The heatmap of patients’ general information and prevalence of emotional disorders

We exported 5813 patient data points from the hospital information system (HIS). 775 patients had sleep disturbances, and 109 had a diagnosis of chronic pain. In addition, 179 patients who underwent operations more than twice during 2015–2020 were excluded. Subsequently, 4571 patients meeting the inclusion and exclusion criteria were included. Flow charting was illustrated in Fig. 2. Their ages ranged from 18 to 95 years old, and the mean age was 52.25 ± 14.85 years. A total of 2436 males accounted for 52.2%. The education of 2506 (53.7%) was junior high school and below. There were 579 (12.4%) with a high school education and 871 (18.6%) with an associate degree. The remaining 715 (15.3%) were undergraduate or above. Regarding marital status, majority of married patients were 4,122 (88.2%). There were 365 (7.8%) unmarried and 184 (3.9%) divorced or widowed patients. The diseased region included cervical (1508, 32.3%), thoracic (170, 3.6%), lumbar (2813, 60.2%) and other parts (180, 3.9%). Others included multi-segment scoliosis and sacrococcygeal disease. The visualization of the patient’s general information was shown in Fig. 3.

### Emotional disorder prevalence

In this study, a total of 539 (11.5%) people developed emotional disorders, of which 319 (6.8%), 151 (3.2%) and 69 (1.5%) had mild, moderate mood and severe emotional disorders, respectively. The details of the emotional disorders of the patients are shown in Fig. 3.

### Effect of multidisciplinary-based psychological management

This study compared the baseline data of patients before and after the implementation of multidisciplinary-based psychological management. The differences between the two groups were statistically significant. Therefore, PSM was used to match the baseline data of the two groups of patients to create a quasi-experimental design. 2107 pairs were matched after PSM. The baseline data and the incidence of emotional disorders in patients before and after PSM were shown in Tables 2 and 3, respectively. The results illustrated that the incidence of emotional disorders in patients dropped down after implementation of psychological management after PSM (*P*<0.001). In the control group, 283 people (13.5%) developed emotional disorders. Of these, 174 (8.3%) had mild emotional disorders, 75 (3.6%)) had moderate emotional disorders, and 34 (1.6) had severe emotional disorders. While 211 (10.0%) people had emotional disorders in the intervention group, of which the mild, moderate and severe emotional disorders were 118 (5.6%), 65 (3.1%) and 28 (1.3%), respectively. Meanwhile, scores of HEI in the intervention group were also heightened compared with those in the control group (*P*<0.001). The severity of emotional disorders was also alleviated with statistical significance (*P* = 0.010).

**Table 2:** The baseline and emotional disorders of the two groups of patients before PSM

Variables	Before implementation *N* = 2226	After implementation *N* = 2445	SMD	*P*
Age (‾x ± s)	51.72 ± 14.93	52.74 ± 14.77	0.069	0.020^a)^
Gender (n, %)				0.006^b)^
Male	1208 (54.3)	1228(50.2)		
Female	1018 (45.7)	1217 (49.8)	0.081	
Education (n, %)				0.002^c)^
Junior high and below	1242 (55.8)	1264 (51.7)	0.082	
Senior high	274 (12.3)	305 (12.5)	0.006	
Associate degree	402 (18.1)	469 (19.2)	0.028	
Undergraduate and above	308 (13.8)	407 (16.6)	0.078	
Marital status (n, %)				0.011^b)^
Married	1986 (89.2)	2136 (87.4)	0.056	
Unmarried	172 (7.7)	193 (7.9)	0.075	
Divorced or Widowed	68 (3.1)	116 (4.7)	0.083	
Diseased region (n, %)				<0.001^b)^
Cervical	728 (32.7)	780 (31.9)	0.017	
Thoracic	88 (4.0)	82 (3.4)	0.032	
Lumbar	1338 (60.1)	1475 (60.3)	0.004	
Others	72 (3.2)	108 (4.4)	0.063	
HEI score (m, IQR)^d)^	3 [1, 6]	2 [0,5]		<0.001^c)^
Emotional disorder (HEI>8) (n, %)	301 (13.5%)	238 (9.7%)		<0.001^b)^
Emotional disorders grades				<0.001^c)^
Mild	185 (8.3%)	134 (5.5%)		
Moderate	81 (3.6%)	70 (2.9%)		
Severe	35 (1.6%)	34 (1.4%)		

Notes: a): *t* value, b)*χ*^*2*^ value, c) *Z* value, d) m: medians; IQR: interquartile ranges

**Table 3:** The baseline and emotional disorders of the two groups of patients after PSM

Variables	Before implementation *N* = 2101	After implementation *N* = 2101	SMD	*P*
Age (‾x ± s)	52.38 ± 14.86	51.96 ± 14.53	0.029	0.315^a)^
Gender (n, %)				0.537^b)^
Male	1090 (51.9)	1110 (52.8)		
Female	1011 (48.1)	991 (47.2)	0.019	
Education (n, %)				0.254^c)^
Junior high and below	1134 (54.0)	1118 (53.2)	0.016	
Senior high	269 (12.8)	258 (12.3)	0.015	
Associate degree	391(18.6)	393 (18.7)	0.003	
Undergraduate and above	307 (14.6)	333 (15.8)	0.033	
Marital status (n, %)				0.669^b)^
Married	1876 (89.3)	1858 (88.4)	0.029	
Unmarried	158 (7.5)	169 (8.0)	0.019	
Divorced or Widowed	67 (3.2)	74 (3.5)	0.017	
Diseased region (n, %)				0.892^b)^
Cervical	691 (32.9)	682 (32.5)	0.009	
Thoracic	73 (3.5)	75 (3.6)	0.005	
Lumbar	1265 (60.2)	1263 (60.1)	0.002	
Others	72 (3.4)	81 (3.9)	0.027	
HEI score (m, IQR)^d)^	3 [1, 6]	2 [0,5]		<0.001^c)^
Emotional disorder (HEI>8) (n, %)	283 (13.5)	211 (10.0)		0.001^b)^
Grades of emotional disorder				0.010^c)^
Mild	174 (8.3)	118 (5.6)		
Moderate	75 (3.6)	65 (3.1)		
Severe	34 (1.6)	28 (1.3)		

Notes: a): *t* value, b)*χ*^*2*^ value, c) *Z* value, d) m: medians; IQR: interquartile ranges

## Discussions

Our findings demonstrated that 11.5% of patients had emotional disorders during spine surgery. This was close to the result of Bekeris et al., who reported that the incidences of anxiety and depression were 6% and 11.2% [23]. Prior study also found the incidence of emotional disorders in spine surgery were higher by using the Patient Reported Outcomes Measurement Information System (PROMIS) screening measures [8]. Although many studies focused on the occurrence of emotional disorders in patients during the perioperative period, the latest research verified that emotional disorders of a small number of patients were difficult to resolve and even developed a new depressive phenotype with the passage of postoperative time [24, 25]. Hence, it is particularly important to manage the emotions of perioperative patients scientifically and effectively. It is well known that an individual’s psychological state was affected by a variety of factors. A previous integrative review confirmed that pain, the need for information, disability, and workforce were factors associated with emotional disorders [12]. Factors influencing new-onset emotional disorders in patients undergoing spinal fusion surgery included long-term opioid using (OR: 1.31–2.93, *P* < 0.01), female sex (OR: 1.25–1.67, *P* < 0.01), longer hospital stay (OR: 1.05–1.08, *P* < 0.01) and readmission within 6 months after surgery (OR range 1.31 to 1.68, *P* < 0.01) [23]. The emotional disorder level increased as preoperative anesthetic uses increased as well [26]. Studies have shown that a single psychological intervention program was less effective than a multimodal psychological intervention program [27]. Hence, setting up various targeted interventions for the aforementioned factors may be able to control the occurrence of emotional disorders in spinal surgery patients.

Notably, patients’ emotional disorders were effectively alleviated by implementing a multidisciplinary-based psychological management. Similar research results were illustrated from the studies of Pennington et al. [22]. The aforementioned literature systematically reviewed the intervention programs and effects of multiple ERAS procedures in spine surgery and mentioned that multidisciplinary-based protocols had mostly followed patient education, blood glucose control, analgesia programs, fluid management, and nutritional management. Multidisciplinary-based management specifically for the psychology of perioperative patients were rare. Furthermore, surgeons might ignore the psychological problems of patients readily [28]. A survey of North American spine surgeons showed that they did not actively measure patients’ awareness of emotional disorders, and only 33.3% of them took the initiative to evaluate patients [28]. A total of 20.3% of spine surgeons preferred to deal with preoperative anxiety by cultivating the nurse–patient therapeutic relationship. Most spine surgeons did not realize their key role in the psychological management of patients. Meanwhile, they believed that this work should mainly allocate by anesthesiologists and patients themselves. In the circumstances, the unity of perception of these entities could support shared decision-making [29].

The interdisciplinary team’s defined roles in the cooperative psychological care of patients were highlighted in our psychological management. A sensible use of medical resources was targeted intervention by medical professionals from many disciplines for individuals with varying degrees of emotional disorders. The improvement of the intervention effect was also facilitated by consideration of elements at all levels during the intervention. In contrast to earlier research, ours placed a greater emphasis on the roles played by each member of the multidisciplinary team as well as the treatment of patients undergoing spine surgery who had varying degrees of emotional disturbances. In order to help with the design of psychological support during spine surgery, we want to offer some references. The team members’ total efforts improved as a result of the lead of Sunshine Angel, a special nurse who served as the key identity and organized the collaboration between several disciplines.

Clinicians are increasingly forced to negotiate an ever-changing healthcare landscape in which the focus of patient care must be delicately balanced between a value-based model that offers high-quality treatment while being accessible and feasible [30]. For this model to be successful, efforts must be aligned across multiple professionals to optimize outcomes and improve patient progress across the continuum of care [31]. The nurse-led management has demonstrated its superiority and cost-effective in several studies [32–34]. For example, a meta-analysis showed that nurse-led psychological interventions were effective in improving patients’ emotional disorders (mean difference: -5.51, 95% CI: -10.68, -0.34) [35]. Nurse-led clinical managements were initially used as a means of controlling rising healthcare costs, not only addressing health care shortages and needs, but also reducing the working hours of junior doctors [36]. Its development has been driven by health policies and the demand for health care, while being supported by the development of the nursing profession [37].

This study fully demonstrated that the leading role of nurses in psychological management could make management more executable. Nurse-led psychological interventions also availed the maintenance of the nurse–patient relationship.

The incidence of emotional disorders reported in this study could arouse clinical staff’s attention to the emotional problems of patients. In addition, this study also reported the effectiveness of Multidisciplinary-based management and the specific content of the program. By showing a more mature psychological management protocol, it was hoped that more clinical staffs could be enlightened in the psychological management of spinal surgery patients during the perioperative period. Finally, the disclosure of this protocol aimed to provide a reference for how multidisciplinary cooperation in improving one clinical outcome could be achieved. It also suggested what responsibilities each department could undertake in multidisciplinary and multimodal management.

Limitations: There were some limitations in our study. First, there were fewer variables that could be included in the PSM, and more confounding factors should be controlled to achieve a higher quality quasi-experimental study in the future. Because we can only get HEI scores after surgery and we only have access to restricted data from HIS. Secondly, more indicators should be added to verify the effect of the management implementation. Due to the single outcome measure, the strength of verifying that the implementation improved emotional disorders directly was limited. It was not clear whether the implementation would improve other indicators, such as pain, to improve emotional disorders. In the future, the relationship between these outcome measures and emotional disorders could be integrated by constructing a structural equation model. Moreover, patients who were diagnosed with different spine disease had various emotional disorder grade probably. Hence, we could conduct further research on the association of spine disease region and emotional disorders.

## Conclusions

Our study used a retrospective PSM controlled study to confirm that multidisciplinary-based psychological management was able to reduce the incidence and improve the severity of perioperative emotional disorders in spine surgery patients. The prevalence of emotional disorders was 11.5% during the perioperative period. The perioperative psychological management of patients requires multidisciplinary cooperation. It was of vital importance that surgeons and nurses clearly understood their responsibilities. In this way, a good doctor-nurse-patient relationship would be maintained while managing the patient’s psychology.

### Electronic supplementary material

Below is the link to the electronic supplementary material.

Supplementary Material 1

## Data Availability

The data that supports the findings of this study are available from the corresponding author upon reasonable request.
